# A comparative study of the prevalence of myopia and behavioral changes in primary school students

**DOI:** 10.1186/s12886-022-02594-6

**Published:** 2022-09-18

**Authors:** Haishao Xiao, Dandan Jiang, Yanhui Wang, Bing Sun, Chunchun Li, Yaoyao Lin, Linjie Liu, Xiaoqiong Huang, Balamurali Vasudevan, Yanyan Chen

**Affiliations:** 1 School of Optometry and Ophthalmology, Wenzhou Medical University, Wenzhou, China; 2 The Eye Hospital, Wenzhou Medical University, Wenzhou, China; 3 National Clinical Research Center for Ocular Diseases, Wenzhou, China; 4 College of Optometry, Midwestern University, Glendale, AZ USA

**Keywords:** Primary school children, Myopia prevalence, Ocular biological parameters, Behaviors

## Abstract

**Objective:**

To analyze the changes in the prevalence of myopia and its relation to ocular biological parameters, and behaviors among primary school students in China, and understand the prevention and control of myopia.

**Methods:**

Cross-sectional surveys were performed on 7–9-year-old children in the yrs. 2012 and 2019. In addition, spherical equivalent refraction (SER), axial length (AL), and AL/corneal radius ratio (AL/CR ratio) were collected without cycloplegia. Participants completed detailed questionnaires on behavior related to myopia.

**Results:**

Data was collected on 623 children (8.02 ± 0.57 years old) in 2012 and 536 students in 2019 (8.09 ± 0.65 years old). The prevalence of myopia was 37.7% in 2012 and 39.9% in 2019. The SER was -0.25 (0.92) D in 2012 and -0.25 (1.25) in 2019. There was no statistical difference in the prevalence of myopia and SER over the 7 years (all *P* > 0.05). In 2019, the prevalence of myopia among girls demonstrated an increasing trend (33.8% vs. 37.8%), but there was no statistical difference (*P* > 0.05). The mean AL and AL/CR ratio of boys were decreasing (all *P* < 0.05). The proportion of children reading more than 2 h and using digital devices for more than 2 h per day after their classes in the 2019 group both decreased (all *P* < 0.05). However, the proportion of activities performed outdoors for more than 2 h./day decreased significantly (*P* = 0.001).

**Conclusion:**

Compared with 2012, the prevalence of myopia in primary school students in 2019 was under control, which may be related to the improvement of children's near-work behavior, but there was the problem of insufficient outdoor activity time. In terms of ocular biological parameters, the risk of myopia for boys in 2019 was lower.

**Supplementary Information:**

The online version contains supplementary material available at 10.1186/s12886-022-02594-6.

## Introduction

Myopia has become a major public health issue worldwide. Global myopia prevalence has reached 22.9%, and it has been predicted that approximately half of the world’s population will have myopia, and about 9.8% of the population will have high myopia by 2050 [1]. Myopia is a common cause of vision loss that leads to poor quality of life with myopic complications. During the emmetropization process, many factors influence the growth of the eye and the refractive error progression. including ocular biological parameters [2], and behaviors like environmental activities [3]. Among these factors, the ocular axis is the most prominent. AL increases as children move from farsightedness to orthophoria and then to myopia [4]. Myopia results when the growth of AL causes the retina to shift back so that the focus of parallel light rays falling into the eye falls in front of the retina [5]. When AL ≥ 26 mm, it greatly increases the risk of serious complications later in life, including the tessellated retina, retinal detachment, subretinal neovascularization, and glaucoma [6].

Asian countries, especially those of the Chinese population, may be more susceptible to myopia in comparison to Western countries [7]. Wenzhou, located in the south of China, is an important regional center in southeastern Zhejiang Province. It is also the only demonstration city of myopia management for teenagers in China [8]. A systemic review released that the prevalence of myopia in Chinese children has increased significantly in recent years [9]. The aim of the present investigation was to study the changes in myopia prevalence, ocular biological parameters, and behaviors related to myopia among children from 2012 to 2019.

## Methods

### Study population

This study was designed as a school-based investigation using random cluster sampling of the children from the Lucheng district of Wenzhou, southeastern China, and three schools were selected randomly. These public schools were in an urban area with a similar campus environment, curriculum, and socioeconomic status. A total of 629 students between the ages of 7–9 years participated in March 2012, and another 538 students between the ages of 7–9 years participated in May 2019. Subjects with a history of any ocular diseases or atropine eye drops or use of orthokeratology lens wear were excluded. The surveys were performed following informed consent from the subjects and one of their parents in 2012. This study was approved by the Ethics Committee of the Eye Hospital of Wenzhou Medical University (approval number: KYK [2014] 3) in 2014 and followed the tenets of the Declaration of Helsinki.

### Eye measurements

Spherical equivalent refraction (SER) and ocular biometric parameters were measured in the years 2012 and 2019. Topcon RM-800 autorefractor (Topcon, Tokyo, Japan) was used to measure refraction three times in each eye to determine an average refractive error without cycloplegia. Spherical power and cylindrical power were recorded. The ocular biometric parameters included axial length (AL), and horizontal and vertical corneal curvature (K1, K2). The ocular biometric parameters were checked by the IOL Master (version 5.4,Carl Zeiss Meditec AG, Jena, Germany) and the Lenstar LS900 Biometer (version 1.1, Haag-Streit AG, Koeniz, Switzerland). A comparison study between both these devices demonstrated a high correlation between them, and the results can be cross-referenced for AL and average K [10].

### Questionnaires

A new questionnaire was developed for this study to understand the behaviors related to the myopia of children (Supplementary file). Some of the questions included age; sex (boys/ girls); average hours per day spent reading after school in the last three months (≤ 1 h/ > 1 and ≤ 2 h/ > 2 h); use of digital devices after school (≤ 1 h/ > 1 and ≤ 2 h/ > 2 h) and participation in outdoor activities (≤ 1 h / > 1 and ≤ 2 h/ > 2 h). The survey also gathered data on the choice of activity during recess breaks between classes (doing homework/ taking activities inside of the classroom/ taking activities outside of the classroom). All students in 2012 and 2019 completed the same questionnaire. Because the children in this study are young, their cognition is limited. Therefore, the questionnaire was completed with the help of parents.

### Definitions

Myopia was defined as SER of ≤ -0.50 diopter (D) which is similar to that used by most epidemiologic researches [11].

Refractive error data for both the eyes of children were highly correlated (2012: Pearson correlation (r) = 0.900, *P* < 0.001; 2019: r = 0.880, *P* < 0.001), so only the right eye data were reported. SER was calculated as the sum of the full spherical power and half of the cylindrical power. The formula to calculate the radius of corneal curvature (CR) was as follows: CR = 1000 (n2-n1)/K. K (average K) = (K1 + K2)/2. N1 (refractive index of air) = 1.0000. N2 (refractive index of cornea) = 1.3375.

### Statistical analysis

Statistical analysis was performed using SPSS (version 24.0). SER was reported as median with interquartile for unnormal distributions. They were compared among groups using Mann–Whitney U test. All ocular biometry parameters were reported as mean ± standard deviations based on the normal distribution with the comparison between groups by the independent-sample student's t-test. Hierarchical data was presented as the number with percentage [n (%)] and analyzed by Mann–Whitney *U* test among groups. Categorical data were expressed in n (%) with the *χ*2 test for intergroup comparison. The changing trend of the prevalence of myopia and ocular biological parameters in the two groups were analyzed by logistic regression or linear regression. All statistical tests were two-sided, and a value of *P* < 0.05 was considered significant.

## Results

Table 1 demonstrates a summary of the data among the children. Among the 1167 children who were enrolled separately in 2012 and 2019, eight children were excluded (4 subjects with ocular diseases and 2 subjects with incomplete questionnaire data in 2012, 2 subjects with orthokeratology lens in 2019). The data of 1159 (99.3%) children [mean (SD) age, 8.05 (0.61) years] was used in the analysis. Among them, 623 children (8.02 ± 0.57 years old) were enrolled in 2012 and another 536 children (8.09 ± 0.65 years old) were enrolled in 2019. There was no statistical difference in the ages between the two groups (*t* = -1.893, *P* = 0.059). The overall prevalence of myopia was 38.7%, and the overall SER was -0.25 (1.120) D. There was no statistical difference in the prevalence of myopia and SER over the 7 years (all *P* > 0.05). However, the AL and AL/CR of children decreased in 2019 (AL: 23.29 ± 0.83 mm vs. 23.12 ± 0.80 mm, *P* = 0.001; AL/CR: 2.98 ± 0.11 vs. 2.96 ± 0.09, *P* = 0.003). The comparison of SER and AL (AL/CR) between two groups of myopia children or non-myopia children was presented in Supplementary Table 1. The SER of non-myopia children had statistical difference between the two groups (2012: 0.05 [0.58] D vs. 2019: 0.13 [0.63] D, *P* = 0.008) as well as the SER of myopia children (2012: -1.00 [2.44] D vs. 2019: -1.25 [1.13] D, *P* = 0.001). In addition, The AL/CR of non-myopia children in 2019 group was lower than that in 2012 group (2.92 ± 0.07 vs. 2.94 ± 0.09, *P* = 0.002).

**Table 1:** Characteristics of the children

Group	All	2012	2019	*χ*2, *t* or *Z*	*P-value*
**N**	1159	623	536		
Sex (N, %)^*^				0.179	0.672
Boy	624(53.8%)	339 (54.4%)	285 (53.2%)		
Girl	535(46.2%)	284 (45.6%)	251 (46.8%)		
**Age**^**§**^	8.05 ± 0.61	8.02 ± 0.57	8.09 ± 0.65	-1.893	0.059
**Age Group (N, %)**					
7	186 (16.0%)	94 (15.1%)	92 (17.2%)		
8	728 (62.8%)	423 (67.9%)	305 (56.9%)		
9	245 (21.2%)	106 (17.0%)	139 (25.9%)		
Myopia Prevalence (N, %)^*^	(38.7%)	(37.7%)	(39.9%)	0.590	0.442
**SER, M(QR), D**^†^	-0.25(1.120)	-0.25 (0.92)	-0.25 (1.25)	-0.301	0.764
**AL, mean ± SD, mm**^**§**^	23.21 ± 0.82	23.29 ± 0.83	23.12 ± 0.80	3.479	0.001
**AL/CR, mean ± SD**^**§**^	2.97 ± 0.1	2.98 ± 0.11	2.96 ± 0.09	3.029	0.003

*SER* spherical equivalent refraction, *M(QR)* median (interquartile range), *D* diopters

^*^χ2 test

^†^Mann–Whitney U test

^§^independent-sample student's t-test

**Fig. 1:**
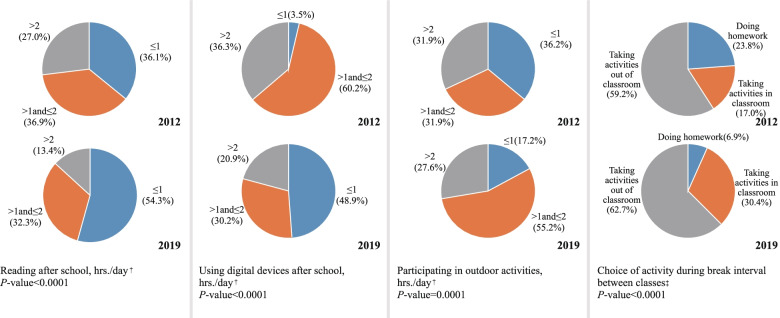
Comparison of behaviors related to myopia between the two cohorts. †, Mann–Whitney U test; ‡, χ2 test

Figure 1 demonstrates the questionnaire results between the two cohorts. In overall students, more than half the subjects chose to perform activities outside the classroom (60.8%) during recess breaks between classes; 29.9% of students spent more than 2 h./day in outdoor activities (data not shown). Compared with the 2012 group, a decreasing proportion of children reading (27.0% vs. 13.4%, *P* < 0.001) and using digital devices (36.3% vs. 20.9%, *P* < 0.001) for a duration of more than 2 h./day after school. There was an increasing proportion of children performing activities inside or outside of the classroom during their break intervals between classes. The proportion of students performing activities outdoors for a duration of > 1 and ≤ 2 h./day increased; however, the proportion of students performing activities for more than 2 h./day decreased (31.9% vs. 27.6%, *P* = 0.001).

In the comparison between the boys and girls, the prevalence of myopia and SER were not significantly different, as presented in Table 2. In 2019, the prevalence of myopia in girls had an increasing trend (33.8% vs. 37.8%, *χ2* = 0.950, *P* = 0.330), but there was no statistical difference. There was no statistical difference in AL or AL/CR in the girls between 2012 and 2019. The AL and AL/CR in the boys in 2019 were shorter than that of boys in 2012 (AL: 23.36 ± 0.79 mm vs. 23.58 ± 0.78 mm, t = 3.551, *P* < 0.001; AL/CR: 2.97 ± 0.09 vs. 2.99 ± 0.11, t = 2.408, *P* = 0.016).

**Table 2:** Comparison of myopia prevalence and ocular parameters between boys or girls of two groups

	**All**	**2012**	**2019**	*χ2*, ***Z* or *t***	***P-value***
**N**	1159	623	536		
Myopia Prevalence (N, %)^*^					
Boys	258 (41.3%)	139 (41.0%)	119 (41.8%)	0.036	0.849
Girls	191 (35.7%)	96 (33.8%)	95 (37.8%)	0.950	0.330
**SER, M(QR), D**^†^					
Boys	-0.25 (1.00)	-0.25 (0.88)	-0.25 (1.25)	-0.022	0.982
Girls	-0.20 (1.12)	-0.25 (0.92)	-0.12 (1.38)	-0.440	0.660
**AL, mean ± SD, mm**^**§**^					
Boys	23.48 ± 0.79	23.58 ± 0.78	23.36 ± 0.79	3.551	< 0.001
Girls	22.89 ± 0.74	22.93 ± 0.75	22.85 ± 0.72	1.351	0.177
**AL/CR**^**§**^					
Boys	2.98 ± 0.10	2.99 ± 0.11	2.97 ± 0.09	2.408	0.016
Girls	2.95 ± 0.10	2.96 ± 0.11	2.95 ± 0.09	1.803	0.072

*SER* spherical equivalent refraction, *M(QR)* median (interquartile range), *D* diopters

^*^χ2 test

^†^Mann–Whitney U test

^§^independent-sample student's t-test

**Table 3:** Comparison of SER and myopia prevalence between the two cohorts according to age

	**Total**	Boys^*^	**SER, M(QR), D**^**†**^	Myopia prevalence (N, %)^*^
**Age 7**				
2012	95	45 (47.9%)	-0.23 (0.98)	30 (31.9%)
2019	91	49 (52.1%)	-0.06 (1.19)	30 (32.6%)
*** χ***2 or *Z*		0.780	-1.525	0.010
* P-value*		0.377	0.127	0.919
**Age 8**				
2012	423	237 (56.0%)	-0.25 (1.00)	159 (37.6%)
2019	305	159 (52.1%)	-0.25 (1.25)	122 (40.0%)
*** χ***2 or *Z*		1.085	-0.115	0.435
* P-value*		0.298	0.908	0.510
**Age 9**				
2012	106	57 (53.8%)	-0.29 (0.67)	46 (43.4%)
2019	139	76 (54.7%)	-0.25 (1.13)	62 (44.6%)
*** χ***2 or *Z*		0.020	-0.357	0.036
* P-value*		0.888	0.721	0.850

*SER* spherical equivalent refraction, *M(QR)* median (interquartile range), *D* diopters

^*^χ2 test

^†^Mann–Whitney U test

**Table 4:** Multivariate regression analyses to estimate the changing trend of prevalence of myopia, SER, AL, and AL/CR with years of test

		**Non-adjusted**		**Model I**		**Model II**	
	Year	*OR* or *β*, (95%CI)	*P-value*	*OR* or *β*, (95%CI)	*P-value*	*OR* or *β*, (95%CI)	*P-value*
Myopia prevalence*	2012	Reference					
	2019	1.050 (0.631, 1.748)	0.850	1.083 (0.853, 1.374)	0.514	1.045 (0.776, 1.408)	0.771
**SER**†	2012	Reference					
	2019	-0.049 (-0.557, 0.246)	0.447	-0.015 (-0.180, 0.106)	0.611	-0.033 (-0.242, 0.079)	0.320
**AL**†	2012	Reference					
	2019	-0.064 (-0.317, 0.103)	0.316	-0.103 (-0.256, -0.081)	** < 0.001**	-0.097 (-0.259, -0.061)	**0.002**
**AL/CR**†	2012	Reference					
	2019	-0.088 (-0.053, 0.009)	0.170	-0.095 (-0.031, -0.008)	**0.001**	-0.104 (-0.034, -0.008)	**0.001**

The model I is adjusted for age and sex; the Model II is adjusted for age, sex, and behaviors related to myopia. *, logistic regression; †, linear regression

In comparison to activities related to myopia between the boys and girls, a decreasing proportion of students who spent their time reading and using digital devices for a duration of more than 2 h./day after school were observed in both boys and girls (all *P* < 0.05). During the recess break intervals between classes, the proportion of boys performing activities inside or outside of the classroom was increasing, similar to that of the girls (all *P* < 0.001). Among the boys, the proportion of performing activities outdoors for a duration of > 1 and ≤ 2 h./day increased (28.6% vs. 53.7%), but the proportion of more than 2 h./day decreased (33.1% vs. 30.2%, *P* = 0.003). Supplementary Table 2 shows the details of the questionnaire results.

**Fig. 2:**
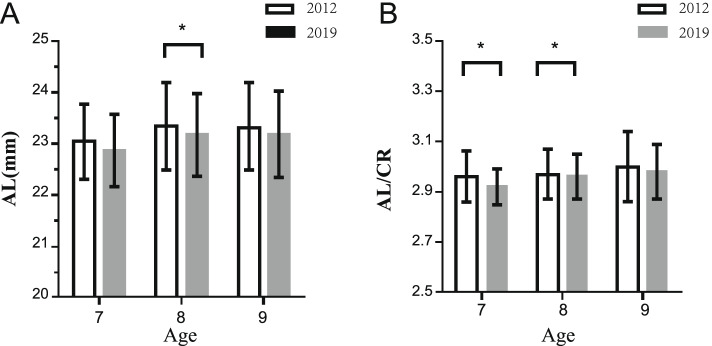
Comparison of ocular biometric parameters among the two populations according to age. A shows the comparison of AL. B shows the comparison of the AL/CR ratio. *, *P* < 0.05

Figure 2 demonstrates a comparison of ocular biometric parameters among the two populations according to age. Compared with the 2012 group, the AL of 8-year-old children in 2019 group decreased (23.34 ± 0.85 mm vs. 23.17 ± 0.80 mm, *t* = 2.825, *P* = 0.005), but there was no change in 7-year-old or 9-year-old children (7-year-old: 23.04 ± 0.73 mm vs. 22.87 ± 0.69 mm, *t* = -1.629, *P* = 0.105; 9-year-old: 23.30 ± 0.81 mm vs. 23.19 ± 0.84 mm, *t* = 1.005, *P* = 0.316). The AL/CR of 7-year-old and 8-year-old children in 2019 group were lower than that in 2012 group (7-year-old: 2.92 ± 0.07 vs. 2.96 ± 0.10, *t* = 2.745, *P* = 0.007; 8-year-old: 2.96 ± 0.09 vs. 2.97 ± 0.10, *t* = 2.046, *P* = 0.041), but there was no change in 9-year-old children **(**2.98 ± 0.11 vs. 3.00 ± 0.14, *t* = 1.375, *P* = 0.170).

Table 3 demonstrates the comparison of the SER and myopia prevalence in both populations according to age. There was no statistical change in SER or myopia prevalence in children aged 7, 8 or,9 years old (all *P* > 0.05).

In comparison with the 2012 group, the proportion of 7, 8, and 9-year-old children in the 2019 group who spent more than 2 h reading and more than 2 h using digital devices after school decreased (all *P* < 0.05). During the break intervals between classes, the proportion of children of all ages performing activities inside or outside of the classroom increased (*P* < 0.05). Supplementary Table 3 shows the details of the questionnaire results.

After adjusting sex, age, and behaviors related to myopia, multivariate regression analysis demonstrates that the prevalence of myopia and SER were not related to the year of investigation, as presented in Table 4. However, compared with the 2012 group, the AL and AL/CR of the children in 2019 was lower [AL: *β* = -0.097, 95% CI (-0.259, -0.061), *P* = 0.002; AL/CR: *β* = -0.104, 95% CI (-0.034, -0.008), *P* = 0.001].

## Discussion

The main finding of the present study was that there were no changes in the prevalence of myopia or SER among primary school students, but the AL and the AL/CR of the boys sub-group decreased. Children's near-work behavior had improved, but there was the problem of insufficient outdoor activity time.

In November 2019, the Wenzhou government disclosed the findings of their myopia survey that was performed with millions of primary and secondary school students in Wenzhou and the overall prevalence of myopia had decreased by 1.45%, which is consistent with the results of the present study [12]. Besides, the results from the present study are similar to the challenges faced by myopic children in Germany and Australia in recent years. The myopia prevalence of children and adolescents aged 0 to 17 years in Germany from 2003 to 2017 had almost not changed (2003–2006 vs. 2014–2017: 11.6% vs. 11.4%) [13]. In another study, no change was found in the prevalence of myopia in students aged 6–15 in Australia from 2014 to 2018 (3.5% vs. 4.4%) [14].On the contrary, in other areas of China, the prevalence of myopia in children has increased in recent years. The prevalence of myopia among 15-year-old school-age children increased from 55.95% in 2005 to 65.48% in 2015 in the Haidian district of Beijing [15]. In Fenghua city, China, the prevalence of myopia among high school students increased from 79.5% in 2001 to 87.7% in 2015 [16]. In Western China, the prevalence of myopia in children increased every year, and the incidence of myopia was as high as 10.6% [17]. A meta-analysis shows that after 2008, the prevalence of myopia among 7 to12-year-old children in China increased from 25.3% to 32.8%, while that of 16 to 18-year-old children increased from 48.4% to 58.7% [9].

However, in the present study, in comparison with the 2012 group, the myopia prevalence of girls in the 2019 group demonstrated an increasing trend (33.8% vs. 37.8%). Although there was no statistical difference, girls’ myopia progression should be watched more carefully in the future. In addition, the age-normal magnitude of hyperopia from the International Myopia Institute (IMI), is estimated to be + 0.50 D for ages 7 to 8 years, and + 0.25 D for ages 9 to 10 years [18]. In our study, the SER of children in 2019 (7 years old: -0.06D; 8 years old: -0.25D; 9 years old: -0.25D) had not reached the proposed IMI standards, even if the magnitude of hyperopia of non-myopia children in 2019 group was better than that in 2012 group (2012: 0.05D vs. 2019: 0.13D). Therefore, although the prevalence of myopia among children in this study had been effectively under control, their hyperopia reserve needs to be improved to prevent the occurrence of myopia.

The mean AL of children in the 2019 group was shorter than that in the 2012 group, which is mainly reflected in the comparison of AL among boys in the two groups. During the development of children's eyeballs, AL maintains irreversible growth. Compared with the cornea and the lens refractive power, AL has a greater impact on ocular refraction. The main reason is that when AL exceeds the normal threshold, other refractive components such as cornea and lens are unable to match, which will possibly lead to myopia. Therefore, the risk of myopia development in boys in the 2019 group may be less than that of boys in the 2012 group.

Compared with the 2012 group, the AL/CR of 7-year-old and AL of 8-year-old in the 2019 group were lower, but the myopia prevalence and SER were not changed. According to the study of Hashemi et al. [9, 19], with AL/CR increasing by 0.1, the ocular refraction increases by -1.21D. He et al. [9, 20] found that as AL/CR increases by 0.1, the progress of myopia was about -1.07D. AL/CR is helpful in the diagnosis of myopia. Therefore, the results of the present study suggest that SER of 7-year-old and 8-year-old children in 2019 may be more inclined to emmetropia than the children of the same age in 2012. The difference was not observed in this study and it may be due to the limited sample size. In addition, multiple regression analysis demonstrates that the survey year was not a risk factor for the prevalence of myopia in children, nor was it associated with SER. However, in comparison with the 2012 group, the children in the 2019 group had shorter AL and lower AL/CR. Given the importance of AL and AL/CR for ocular refraction, the risk of myopic complications for children in the 2019 group may be lower than that of the children in the 2012 group.

Compared with the 2012 group, the proportion of children reading more than 2 h and using digital devices for more than 2 h per day after their class in the 2019 group has decreased. Multiple studies show that increasing near-work time is a risk factor for children's myopia. Ding et al. [21] found that the difference in near-work time can influence the difference in SER between twins (*β* = -0.11[D]/h). A survey of children in grades 4–6 in Taiwan indicates that near working for more than one hour per day increased the risk of myopia (*OR* = 1.26) [22]. The present study found that the proportion of children in the 2019 group performing activities in or out of the classroom during recess increased. Recent evidence has shown that long-term continuous near work is a dangerous factor for children's myopia [23, 24]. The mechanism may be due to the accumulation of temporary myopia induced by near work that leads to persistent retinal defocus, which eventually makes AL longer and myopia progression [25]. In this study, compared with the children in 2012, the children in 2019 have less near-work time; more students chose to leave their desks during recess to perform activities, which indicates that the children have improved their behavior related to myopia. The control of myopia may be related to it.

In this study, the control of myopia status and improvement of behaviors among children in Wenzhou from 2012 to 2019 may be related to the prevention and control of myopia by the Wenzhou government in recent years. As a national pilot city for myopia prevention and control, the Wenzhou Municipal Government has actively carried out a general survey of myopia on campuses and regulated the use of digital devices among children; its near-sight prevention policies have a wide coverage and high requirements for implementation.

Nevertheless, the proportion of children in the 2019 group who spent more than two hours decreased. A large number of studies have found that increasing outdoor activities can reduce the risk of myopia and delay the progression of myopia [26–28]. Only performing outdoor activities for more than 2 h per day or more than 14 h a week can play a protective role in myopia [27]. Outdoor activities may prevent myopia through a variety of ways, of which high light-induced dopamine release is the most prominent [29]. In terms of outdoor activities, this study demonstrates that children's outdoor activity time was insufficient, and it is necessary to increase children's outdoor activity time to prevent myopia.

There are a few limitations of the present study. First, this study investigated only a small population, so a large sample of children should be included in future studies. Second, behavioral data were collected through questionnaires, which may cause recall bias. Despite these limitations, our research provides a basis for evaluating the changes in myopia prevalence and behaviors related to the prevalence of myopia in children in southern China in 2012 to 2019.

## Supplementary Information

**Additional file 1:** 

**Additional file 2:** **SupplementaryTable 1.**Comparison of SER and AL (AL/CR) in myopia or non-myopia between two groups.

**Additional file 3:** **Supplementary Table 2.** Comparison ofbehaviors related to myopia between boys or girls in two groups

**Additional file 4:** **SupplementaryTable 3.**Comparison of behaviorsrelated to myopia between the twoCohorts according to age

## Data Availability

The datasets analyzed in this study are available from the corresponding author (Yanyan Chen, wzcyymail@163.com) upon reasonable request.

## Abbreviations

SER: Spherical equivalent refraction
AL: Axial length
AL/CR ratio: AL/corneal radius ratio
K1: Horizontal corneal curvature
K2: Vertical corneal curvature
CR: Corneal curvature
IMI: International Myopia Institute
