# Supplementary material for: A comparative study of the prevalence of myopia and behavioral changes in primary school students
Source: BMC Ophthalmol. 2022 Sep 18;22:370. doi: 10.1186/s12886-022-02594-6 (PMC9482727; doi:10.1186/s12886-022-02594-6)
Supplement: Supplementary file 1 — Additional file 1: [file 12886_2022_2594_MOESM1_ESM.docx]

***English Version***

**The Wenzhou Epidemiology of Refraction Error (WERE) study Questionnaire**

1. Child’s name:
2. Child’s Sex:
3. Child’s Grade , Class
4. Child’s Date of birth:
5. Do you currently wear orthokeratology lenses?

□ Yes □No

6. Please complete the following questions according to child’s activities.

| Activities | Options | | |
| --- | --- | --- | --- |
| Reading after school, hrs./day | 1. ≤1 | 1. >1 and ≤2 | 1. >2 |
| Using digital devices after school, hrs./day | 1. ≤1 | 1. >1 and ≤2 | 1. >2 |
| Participating in outdoor activities, hrs./day | 1. ≤1 | 1. >1 and ≤2 | 1. >2 |
| Choice of activity during break interval between classes | 1. Doing homework | 1. Taking activities in classroom | 1. Taking activities out of   classroom |

7. Father’s Date of birth:

8. Mother’s Date of birth:

9. Father’s education level:

10. Mother’s education level:

11. Father’s occupation:

12. Mother’s occupation:

13. Did child’s father have the following vision problems?

□Myopia □Hyperopia □Astigmatism □NO

What’s the most myopic or hyperopic refractive of the two eyes?

□<-6.0D □-6.0 to -3.0D □>-3.0D

14. Did child’s mother have the following vision problems?

□Myopia □Hyperopia □Astigmatism □NO

What’s the most myopic or hyperopic refractive of the two eyes?

□<-6.0D □-6.0 to -3.0D □>-3.0D
