# Supplementary material for: A comparative study of the prevalence of myopia and behavioral changes in primary school students
Source: BMC Ophthalmol. 2022 Sep 18;22:370. doi: 10.1186/s12886-022-02594-6 (PMC9482727; doi:10.1186/s12886-022-02594-6)
Supplement: Supplementary file 2 — Additional file 2: SupplementaryTable 1.Comparison of SER and AL (AL/CR) in myopia or non-myopia between two groups. [file 12886_2022_2594_MOESM2_ESM.docx]

**Supplementary Table 1**. Comparison of SER and AL (AL/CR) in myopia or non-myopia between two groups.

| **Group** |  | **2012** | **2019** | ***t* or *Z*** | ***P-value*** |
| --- | --- | --- | --- | --- | --- |
| **Myopia** | **SER, M(QR), D**^†^ | -1.00 (2.44) | -1.25 (1.13) | -3.214 | 0.001 |
|  | **AL, mean±SD, mm^§^** | 26.63±0.88 | 23.54±0.83 | 1.221 | 0.223 |
|  | **AL/CR, mean±SD^§^** | 3.03±0.10 | 3.02±0.09 | 1.230 | 0.219 |
| **Non-myopia** | **SER, M(QR), D**^†^ | 0.05 (0.58) | 0.13 (0.63) | -2.641 | 0.008 |
|  | **AL, mean±SD, mm^§^** | 22.98±0.72 | 22.92±0.68 | 1.183 | 0.237 |
|  | **AL/CR, mean±SD^§^** | 2.94±0.09 | 2.92±0.07 | 3.156 | 0.002 |

SER, spherical equivalent refraction; M(QR), median (interquartile range); D, diopters; ^†^, Mann–Whitney U test; ^§^, independent-sample student's t-test.
