# Supplementary material for: A comparative study of the prevalence of myopia and behavioral changes in primary school students
Source: BMC Ophthalmol. 2022 Sep 18;22:370. doi: 10.1186/s12886-022-02594-6 (PMC9482727; doi:10.1186/s12886-022-02594-6)
Supplement: Supplementary file 3 — Additional file 3: Supplementary Table 2. Comparison ofbehaviors related to myopia between boys or girls in two groups [file 12886_2022_2594_MOESM3_ESM.docx]

**Supplementary Table 2**. Comparison of behaviors related to myopia between boys or girls in two groups

|  | **Boys** | | |  | **Girls** | | |
| --- | --- | --- | --- | --- | --- | --- | --- |
| **Behaviors** | **2012^§^** | **2019^§^** |  |  | **2012^§^** | **2019^§^** |  |
|  | **(n=339)** | **(n=285)** | ***P-value*** |  | **(n=284)** | **(n=251)** | ***P-value*** |
| **Reading after school,** hrs./day^†^ |  |  | **0.001** |  |  |  | **＜0.001** |
| ≤1 | 139 (41.0%) | 159 (55.8%) |  |  | 86 (30.3%) | 132 (52.6%) |  |
| >1 and ≤2 | 126 (37.2%) | 79 (27.7%) |  |  | 104 (36.6%) | 94 (37.5%) |  |
| >2 | 74 (21.8%) | 47 (16.5%) |  |  | 94 (33.1%) | 25 (9.9%) |  |
| **Using digital devices after school,** hrs./day^†^ |  |  | **＜0.001** |  |  |  | **＜0.001** |
| ≤1 | 10 (2.9%) | 141 (49.5%) |  |  | 12 (4.2%) | 121 (48.2%) |  |
| >1 and ≤2 | 186 (54.9%) | 79 (27.7%) |  |  | 189 (66.5%) | 83 (33.1%) |  |
| >2 | 143 (42.2%) | 65 (22.8%) |  |  | 83 (29.3%) | 47 (18.7%) |  |
| **Choice of activity during break interval between classes**^‡^ |  |  | **＜0.001** |  |  |  | **＜0.001** |
| Doing homework | 57 (16.8%) | 17 (6.0%) |  |  | 91 (32.0%) | 20 (8.0%) |  |
| Taking activities in the classroom | 63 (18.6%) | 70 (24.6%) |  |  | 43 (15.1%) | 93 (37.1%) |  |
| Taking activities out of the classroom | 219 (64.6%) | 198 (69.4%) |  |  | 150 (52.8%) | 138 (55.0%) |  |
| **Participating in outdoor**  **activities,** hrs./day^†^ |  |  | **0.003** |  |  |  | 0.160 |
| ≤1 | 130 (38.3%) | 46 (16.1%) |  |  | 95 (33.5%) | 46 (18.3%) |  |
| >1 and ≤2 | 97 (28.6%) | 153 (53.7%) |  |  | 102 (35.9%) | 143 (57.0%) |  |
| >2 | 112 (33.1%) | 86 (30.2%) |  |  | 87 (30.6%) | 62 (24.7%) |  |

^†^, Mann–Whitney U test; ^‡^, χ2 test; ^§^, number (%).
