# Supplementary material for: A comparative study of the prevalence of myopia and behavioral changes in primary school students
Source: BMC Ophthalmol. 2022 Sep 18;22:370. doi: 10.1186/s12886-022-02594-6 (PMC9482727; doi:10.1186/s12886-022-02594-6)
Supplement: Supplementary file 4 — Additional file 4: SupplementaryTable 3.Comparison of behaviorsrelated to myopia between the twoCohorts according to age [file 12886_2022_2594_MOESM4_ESM.docx]

**Supplementary Table 3**. Comparison of behaviors related to myopia between the two Cohorts according to age

| **Behaviors** | **Age 7** | |  | **Age 8** | |  | **Age 9** | |  |
| --- | --- | --- | --- | --- | --- | --- | --- | --- | --- |
|  | **2012^§^** | **2019^§^** | ***P-value*** | **2012^§^** | **2019^§^** | ***P-value*** | **2012^§^** | **2019^§^** | ***P-value*** |
|  | (n=94) | (n=92) |  | (n=423) | (n=305) |  | (n=106) | (n=139) |  |
| **Reading after school,** hrs./day^†^ |  |  | **0.004** |  |  | **＜0.001** |  |  | **0.001** |
| ≤1 | 45 (47.9%) | 62 (67.4%) |  | 147 (34.8%) | 152 (49.8%) |  | 33 (31.1%) | 77 (55.4%) |  |
| >1 and ≤2 | 24 (25.5%) | 19 (20.7%) |  | 149 (35.2%) | 108 (35.4%) |  | 57 (53.8%) | 46 (33.1%) |  |
| >2 | 25 (26.6%) | 11 (12.0%) |  | 127 (30.0%) | 45 (14.8%) |  | 16 (15.1%) | 16 (11.5%) |  |
| **Using digital devices after school,** hrs./day^†^ |  |  | **＜0.001** |  |  | **＜0.001** |  |  | **＜0.001** |
| ≤1 | 6 (6.4%) | 76 (82.6%) |  | 10 (2.4%) | 108 (35.4%) |  | 6 (5.7%) | 78 (56.1%) |  |
| >1 and ≤2 | 47 (50.0%) | 13 (14.1%) |  | 256 (60.5%) | 114 (37.4%) |  | 72 (67.9%) | 35 (25.2%) |  |
| >2 | 41 (43.6%) | 3 (3.3%) |  | 157 (37.1%) | 83 (27.2%) |  | 28 (26.4%) | 26 (18.7%) |  |
| **Choice of activity during break interval between classes**^‡^ |  |  | **0.006** |  |  | **＜0.001** |  |  | **0.001** |
| Doing homework | 18 (19.1%) | 4 (4.3%) |  | 105 (24.8%) | 22 (7.2%) |  | 25 (23.6%) | 11 (7.9%) |  |
| Taking activities in classroom | 25 (26.6%) | 25 (27.2%) |  | 69 (16.3%) | 105 (34.4%) |  | 12 (11.3%) | 33 (23.7%) |  |
| Taking activities out of  classroom | 51 (54.3%) | 63 (68.5%) |  | 249 (58.9%) | 178 (58.4%) |  | 69 (65.1%) | 95 (68.4%) |  |
| **Participating in outdoor**  **activities,** hrs./day^†^ |  |  | **0.006** |  |  | 0.152 |  |  | 0.079 |
| ≤1 | 32 (34.0%) | 0 (0%) |  | 157 (37.1%) | 74 (24.3%) |  | 36 (34.0%) | 18 (12.9%) |  |
| >1 and ≤2 | 34 (36.2%) | 69 (75.0%) |  | 129 (30.5%) | 146 (47.9%) |  | 36 (34.0%) | 81 (58.3%) |  |
| >2 | 28 (29.8%) | 23 (25.0%) |  | 137 (32.4%) | 85 (27.9%) |  | 34 (32.1%) | 40 (28.8%) |  |

^†^**,** Mann–Whitney U test; ^‡^**,** *χ^2^* test; **^§^,** number (%).
